# The antidepressant effects of kaji-ichigoside F1 via activating PPAR-γ/CX3CR1/Nrf2 signaling and suppressing NF-κB/NLRP3 signaling pathways

**DOI:** 10.3389/fphar.2025.1569888

**Published:** 2025-04-16

**Authors:** Maoyang Huang, Faju Chen, Lang Zhou, Qing Zhang, Li Wang, Liangqun Li, Lishou Yang, Ming Gao, Lilang Li, Yu Wang, Juan Yang, Guanping Yao, Qiji Li, Xiaosheng Yang

**Affiliations:** ^1^ State Key Laboratory of Discovery and Utilization of Functional Components in Traditional Chinese Medicine, Guizhou Medical University, Guiyang, China; ^2^ Natural Products Research Center of Guizhou Province, Guiyang, China

**Keywords:** kaji-ichigoside F1, depression, PPAR-γ/CX3CR1/Nrf2, NF-κB/NLRP3, LPS

## Abstract

**Introduction:**

Depression is a mental illness closely associated with neurological damage and is characterised by high rates of suicide and mood changes. As a traditional medicinal plant, *Rosa roxburghii* Tratt has been widely used since ancient times in the *Miao* and *Dong* regions of Southwest China for the relief of sleep disorders, indigestion, anti-inflammation, neurasthenia and neuroprotection. The total triterpenes of *R. roxburghii* were previously found to have certain neuroprotective effects, and whether Kaji-ichigoside F1 (KF1), as its main ingredient, plays a relevant pharmacological role needs to be further investigated.

**Methods:**

Establishment of mouse depression model and BV2 microglia inflammation model using intraperitoneal injection of LPS in mice and LPS stimulated-BV2 microglia, respectively. The antidepressant effects of KF1 were evaluated by forced swim test (FST), sucrose preference test (SPT), tail suspension test (TST) and open field test (OFT). The number of Nissl bodies and apoptotic positive cells in the CA1 region of the hippocampus was observed by Nissl and TUNEL staining. Then, the levels of TNFα, PPAR-γ, TGF-β, and IL-6 cytokines were tested by ELISA kits. Finally, the molecular mechanisms were investigated by Western blotting (WB) and immunofluorescence *in vivo* and *in vitro*.

**Results:**

KF1 dramatically ameliorated LPS-induced depressive like behaviors, neuronal damage, apoptosis, and suppressed the levels of pro-inflammatory cytokines in the serum and hippocampus of mice. Our vitro experiment also showed KF1 significantly reduced cell viability and attenuated apoptosis in LPS-induced BV2 microglia, decreased the mean fluorescence intensity of Caspase-1, TNFα, NF-κB, IL-1β, NLRP3, and Keap1. However, the mean fluorescence intensity of GCLC, GCLM, GST, SOD1, HO-1, and Nrf2 were significantly increased. Finally, Western blot analysis showed that KF1 suppressing the expression of NF-κB/NLRP3 signaling pathway and activating PPARγ/CX3CR1/Nrf2 signaling pathway both *in vivo* and *in vitro*.

**Conclusion:**

In conclusion, these results suggest that KF1 is an effective alleviator of LPS-induced depression-like effects *in vivo* and *in vitro*. These effects were associated with activating PPARγ/CX3CR1/Nrf2 signaling, and suppressing NF-κB/NLRP3 signaling pathways.

## Graphical Abstract

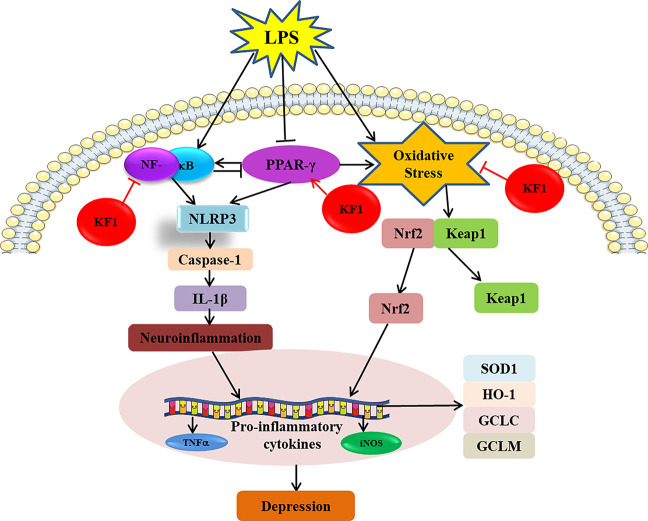

## 1 Introduction

Depression, a high morbidity, recurrent, and fatal mental disorder, is characterized by lasting sadness, disinterest, sleep disturbances, and suicidal tendencies, which seriously impacts patient’s quality of life (Lin et al., 2017; Tao et al., 2021). The traditional antidepressant drugs, such as selective serotonin reuptake inhibitors and tricyclics, are usually taken for weeks or months at a time and have serious side effects in pharmacological treatment (Xia et al., 2023). These deficiencies highlight the urgent need for safer and more effective antidepressants.

Neuroinflammation is characterized by an abnormal immune response in the brains of depressed patients, which is mainly involved in the development of depression through different neurobiological mechanisms (Liu et al., 2024). NLRP3 inflammasome is a multiprotein complex that plays a key role in the inflammatory response in the central nervous system (CNS) (Saad et al., 2024; Zhang et al., 2018). The emergence of depression is closely related to the stimulation of mature NLRP3 inflammasome triggering the activation of Caspase-1, which in turn promotes the maturation and release of pro-inflammatory cytokines. Studies have found the levels of IL-6, NF-κB, TNFα and IL-1β were increased in the blood and brain tissue with depression (Xu et al., 2024). Pro-inflammatory cytokines cause neuronal damage, changes in brain structure, and impaired communication between neurons (Zhao et al., 2024). Inflammation levels return to normal with antidepressant medication in depressed patients, however, they remain elevated in untreated patients. Thus, inflammation levels play an important role in depression.

*Rosa roxburghii* Tratt is classified as the *Rosa* genus in the Rosaceae family, is a famous food and medicine multi-ethnic plant resource in China, which is widely cultivated and used in the *Miao* and *Dong* nationality areas of Guizhou Province (Jain et al., 2024). There is a record in the *Qianshu* of the 29th year of the Kangxi Dynasty that “food has been boring elimination of accumulation”, which has been used as medicine in China for more than 300 years. It has been reported that *R*. *roxburghii* has neuroprotective effects and has been used to treat neurasthenia, sleep disturbances, and indigestion (Chen et al., 2022). Building on traditional knowledge linking herbal remedies to mood regulation, our study has shown that the total triterpenoids from *R. roxburghii* have antidepressant effects (Yang et al., 2021). KF1 is a natural oleanane-type triterpenoid saponin and its content surpasses 0.17 mg/g in *R*. *roxburghii* (Chen et al., 2024). In this study, we attempted to investigate the mechanism of antidepressant effects of KF1 by *in vivo* and *in vitro* experiments, This study investigates the antidepressant mechanism of KF1 through the PPARγ/CX3CR1/Nrf2 and NF-κB/NLRP3 signaling pathways. The aim of this study is to provide a reference for the ethnographic application of *R*. *roxburghii*, especially its characteristic constituent KF1.

## 2 Materials and methods

### 2.1 Reagents

Extraction of Kajiichigoside F1 (KF1): The fruits of Rosa roxburghii (150 kg) were extracted with 95% aqueous ethanol under reflux to yield a crude extract (11.7 kg). This extract was then suspended in water (25 L) and sequentially partitioned with petroleum ether (PE) and ethyl acetate (EAC), affording PE (64.97 g) and EAC (914.5 g) extracts, respectively. The EAC extract (914.5 g) was subjected to silica gel column chromatography, eluted with a dichloromethane/methanol gradient (20:1 to 0:1, v/v), yielding six fractions (Fr.B1−Fr.B6). From fraction Fr.B3, Kajiichigoside F1 was isolated through repeated silica gel column chromatography using dichloromethane/methanol (10:1, v/v) as the eluent, achieving a purity greater than 95%.

The ELISA PPARγ(#20240611) kit was obtained from hua mei biotechnology Co. (Wuhan, China); The ELISA TGF-β (#20231208), IL-6 (#20240621, and TNFα (#20240308) kits were obtained from wuhan sanying Biotechnology Co.(Wuhan, China); The Nissl staining (#20231102), TUNEL staining(#20231201), PI staining(#20240618), LDH(#20231022), BCA(#20221109),and MMP(#20231121) were obtained from solabio ciotechnology Co. (Beijing, China); Anti-caspase-1 (ab207802), COX2 (ab179800), NF-κB (ab32536), IL-β (ab187060), iNOS (ab178945), TNFα (ab183218), NLRP3 (ab263899), GCLC (ab207777), GCLM (ab126704), SOD1 (ab308181), HO-1 (ab189491), Nrf2 (ab62352), Keap1 (ab119403), and PPARγ (ab272718) were obtained from Abcam (Cambridge, USA).

### 2.2 Animals

Adult male C57BL/6J mice weighing 18–22 g (7–8 weeks of age) were used for our experiments, and the animals were a gift from Hangzhou Ziyuan Laboratory Animal Co. Ltd. (Hangzhou, China). All animals were housed individually in standard conditions, which included a 12-h light/dark cycle, 65% humidity, and a temperature of 22°C ± 2 C, with free access to purified water and diet. The protocol approved by the Animal Ethics Committee of Guizhou Medical University was followed for all experimental animals and procedures.

### 2.3 Experimental protocol

**FIGURE 1:**
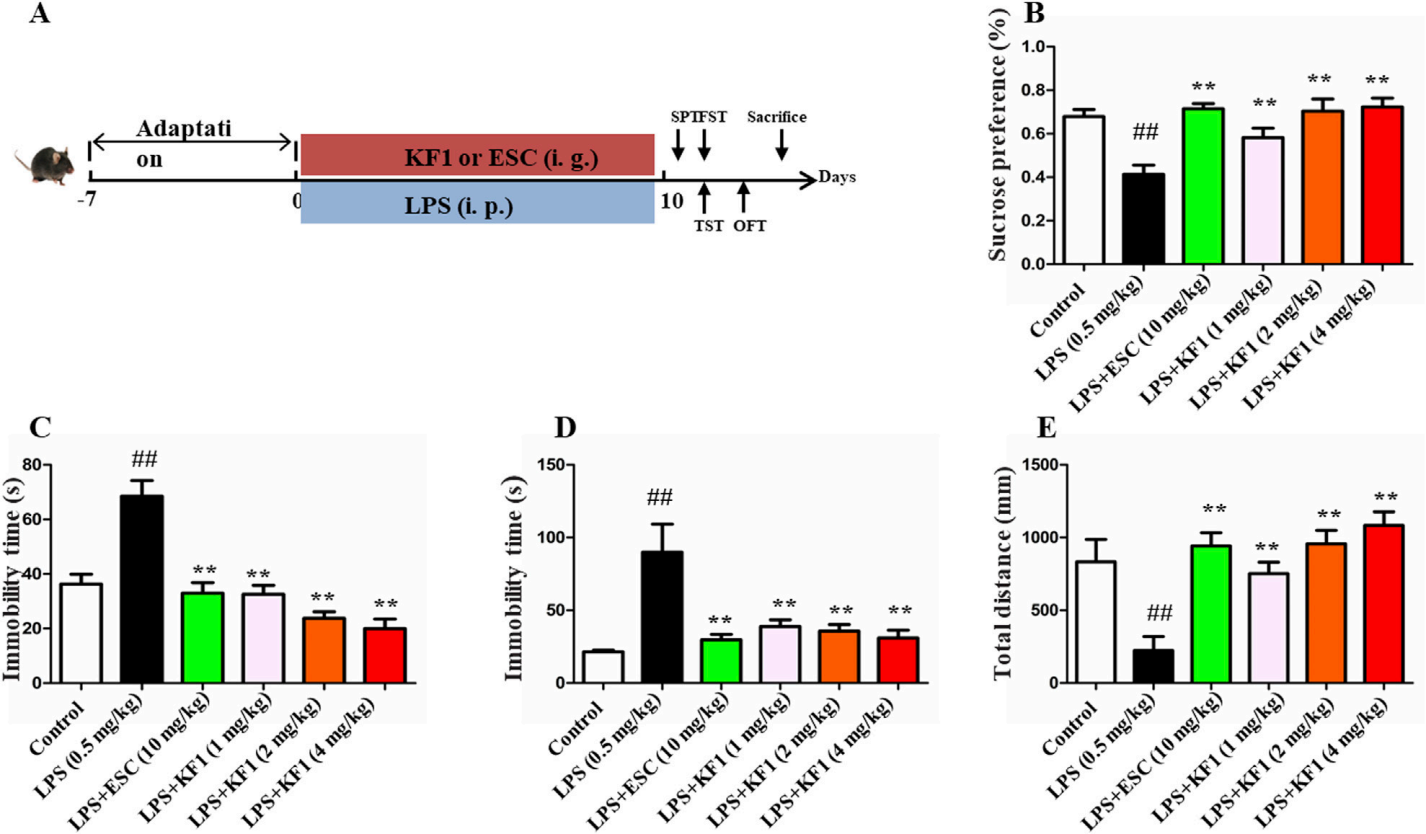
A Figure 1 Antidepressant-like effect of KF1 on SPF, FST, TST, and OFT in LPS induced mice. **(A)** The process of experiment; **(B)** Sucrose preference (%); **(C)** the immobility time in FST; **(D)** the immobility time in TST; **(E)** the total distance in OFT. The data were expressed as mean ± SEM; ^##^ *p <* 0.01, compared with the control group, **p < 0.01, compare with the LPS group.

After the adaptation, the male mice were divided into six groups, each containing 10 mice. The groups were the control group, the LPS (0.5 mg/kg, i.p.) group, the LPS + ESC (escitalopram, 10 mg/kg, i.g.) group, and the LPS + KF1 (1, 2, 4 mg/kg, i.g.) group. KF1 or ESC were orally administered once daily for 14 consecutive days, and LPS modeling was performed intraperitoneally once a day for the subsequent 4 days from the 10th day, as shown in Figure 1A. After 12 h of LPS challenge, the behavioral assessment were carried out to evaluate the behavioral activity (Li Y. et al., 2021). Subsequently, all mice were anesthetized with 1% pentobarbital sodium, blood samples were collected via orbital puncture, and then used to obtain serum layers by centrifuging them for 20 min at 4 °C at a speed of 3500 rpm. Following which hippocampus tissues were stripped with ophthalmic forceps and frozen in liquid nitrogen and stored at −80°C.

### 2.4 Animal behavioral testing

#### 2.4.1 Sucrose preference test

SPT is one of the typical symptoms in mice for assessing dysphoria as a model of depression (Song et al., 2018). Briefly, mice housed in single cages were deprived of purified water and diet for 24 h, then all animals were adapted to two bottles of 1% sucrose solution (w/v) for 12 h, and followed by replacement of tap water 12 h period, respectively. After this adaptation phase, and then given access to two bottles for 12 h (one bottle of purified water, Another bottle of 1% sucrose solution), each bottle changes position every 2 h to avoid a side bias. The sucrose preference is defined as the sucrose consumption/(sucrose + water consumption) × 100%.

#### 2.4.2 Open field test

Previous research indicates that the OFT test is used to assess autonomous movement and depression-like behaviors in lab animals (Lan et al., 2022). The experiment was conducted in a quiet environment and each mouse individually allowed explore freely for 2 min before the test. The total distances of movement in the central area within 4 min were monitored by the video recording system. After each mouse has been tested, the square observation cage was swabbed with a 70% ethanol solution to prevent the peculiar smell avoid affecting the next test.

#### 2.4.3 Forced swimming test

The FST is a traditional technique used to evaluate behavioral despair in mouse models of depression (Sun et al., 2020). Each mouse was individually placed in a clear glass cylinder (30 cm tall, 10 cm in diameter) with water kept at a temperature of 24°C ± 1 °C. All mice were forced placed in the water filled cylinder to swim for 6 min test session, and the last 4 min of immobility times were taped by video.

#### 2.4.4 Tail suspension test

The TST experimental procedure was performed according to the reference consistent with Hiraoka (Hiraoka et al., 2017). In this experiment, each mouse was hung 30 cm above the ground, with about 1.5 cm of its tail secured. The total duration of the experiment was 6 min, and immobility time was noted during the last 4 min.

### 2.5 Cell culture and treatment

BV2 cells, a immortalized murine microglia cell derived from mouse, were purchased from institute of Cell Biology, of Chinese Academy of Sciences Accession (Number: CVCL-0182, Shanghai, China), and were cultured in DMEM (Gibco, USA) with 1% penicillin/streptomycin and 10% fetal bovine serum (FBS, BI, Israel) at 37°C in a 95% air and 5% CO_2_ environment. Subsequent experiments were started using BV2 cells at passages 3–6. Cells were used for experiments when cells density reached approximately 80%. The experiment was divided six groups: control group, LPS (1 μg/mL) group, LPS (1 μg/mL) group + ESC (10 μg/mL), LPS (1 μg/mL) + KF1 (5 μM) group, LPS (1 μg/mL) + KF1 (10 μM) group, LPS (1 μg/mL) + KF1 (20 μM) group. After culturing for 24 h, cells were harvested for further experiments.

### 2.6 Cell viability assay

The MTT assay was used to assess the viability of BV2 cells. In short, BV2 cells at a concentration of 1 × 10^*^5 cells/ml were grown in a 96-well plate and exposed to varying concentrations of KF1, with or without LPS. After 24-h incubation, the cells were treated with an MTT solution (0.5 mg/mL) for 30 min. The medium was then removed, and 200 μL of DMSO was added to each well to dissolve the formazan product. The absorbance was recorded at 570 nm wavelength by multifunctional enzyme labeling, and results of cell survival (%) = (A (experiment) – A (blank))/(A (control) – A (blank)) × 100%.

### 2.7 ELISA assay

The concentration of TGF-β (#20231208, Proteintech, China), PPARγ (#20240611, Cusabio, China), IL-6 (#20240621, Proteintech, China) and TNFα (#20240308, Proteintech, China) in serums and hippocampus supernatants were measured by ELISA kits in accordance with the manufacturer’s instructions. Absorbance was determined using Varioskan LUX at 450 nm.

### 2.8 Nissl staining

The performes and analyses for Nissl staining are same as in previous studies (Chen et al., 2024). To summarize, after the behavioral tests concluded, the mice were heavily anesthetized using chloral hydrate, and three mice from each group were randomly chosen to have their entire brains extracted and preserved in 4% paraformaldehyde for 4 days. The brain tissue fixed with formaldehyde was dehydrated, embedded in paraffin, and sliced into Section 5 µm thick. For Nissl staining, the coronal brain sections were deparaffinized using xylene and rehydrated through a series of graded alcohols. The sections were immersed Nissl staining solution at 37 C for 5 min, washed with distilled water, followed by xylene-based mounting medium to evaluate the degree of neuronal damage in the brains of mice. Lastly, sections of Nissl staining images were observed using a light microscope and count the Nissl positive cells.

### 2.9 TUNEL staining

Tissues were embedded in paraffin as described above. Apoptotic cells were identified by TUNEL staining kit. In brief, the brain sections with repaired antigens underwent incubation with the TUNEL reaction mixture at 37 °C for 60 min. This was followed by three 5-min washes with PBS and 10-min incubation with DAPI. In the end, the brain sections underwent three washes and were observed under a fluorescence microscope (Olympus, Tokyo, Japan). ImageJ software was used to count the TUNEL-positive cells.

### 2.10 Propidium iodide (PI) staining

Cell death was measured using the PI staining assay (Cai et al., 2019). Cells treated with drugs were rinsed using PBS, incubated with DAPI and PI for 5 min in the dark at common temperature. Subsequently, apoptosis was observed using fluorescence microscopy.

### 2.11 Lactate dehydrogenase (LDH)

The quantitative determination of cytotoxicity involved measuring lactate dehydrogenase (LDH) released from damaged cells into the culture medium, according to the manufacturer’s instructions. Absorbance was detected on a microplate reader at 490 nm.

### 2.12 Measurement of mitochondrial membrane potential (MMP)

Mitochondrial transmembrane potential (Δψm) is a marker of mitochondria damage (Zhang et al., 2021). To measure the Δψm, JC-1staining (Invitrogen), BV2 cells were exposed to JC-1 dye (Invitrogen) for 15 min at room temperature, washed three times with PBS, and the MMP was determined by the red/green fluorescence intensity ratio observed under a fluorescence microscope.

### 2.13 Immunofluorescence staining

After drug treatment, BV2 cells in 6-well plates were fixed with 4% paraformaldehyde for 20 min at room temperature and then permeabilized with 0.1% TritonX-100 in PBS for 10 min. Subsequently, cells were cleaned with cold PBS and left in a blocking solution with PBS and 5% BSA for 60 min at room temperature. The cells were incubated with Caspase-1(1:100), TNFα (1:100), IL-1 (1:100), and NLRP3 (1:100), CX3CR1 (1:100), HO-1 (1:100), IL-1β (1:100), Nrf2 (1:100), Keap1 (1:100), and PPARγ (1:100) antibodies at 4 C overnight. After three washings with PBS, incubate with green secondary antibody for 2 h at room temperature. Nuclei were counterstained with DAPI for 5 min and then fluorescent images were obtained by fluorescence microscope.

### 2.14 Western blotting

Collected total proteins from hippocampus tissue and BV2 cells, and quantification of protein concentration with BCA kits (Beyotime, Nanjing, China) following the manufacturer’s guidelines. Western blot tests were conducted and evaluated following the methods of our earlier studies. In summary, equal quantities of denatured proteins were separated using SDS-PAGE and then transferred onto PVDF membranes. The membrane blocked with nonfat dry milk in TBST, and then sequentially incubated with primary antibodys: caspase-1 (1:1000), COX2 (1:1000), NF-κB (1:1000), IL-β (1:1000), iNOS (1:1000), TNFα (1:1000), NLRP3 (1:1000), GCLC (1:1000), GCLM (1:1000), SOD1 (1:1000), HO-1 (1:1000), Nrf2 (1:1000), Keap1 (1:1000), and PPARγ (1:1000) at 4 °C overnight. Following three washes with PBST, the membranes were incubated with a secondary antibody for an hour at room temperature and visualized using the enhanced ECL Plus kit. ImageJ software was employed to analyze the strip grey values.

### 2.15 Statistical analysis

Each study’s data was replicated a minimum of three times, presented as mean ± SEM, and analyzed with GraphPad Prism version five software. One-way ANOVA and Tukey *post hoc* tests were applied to analyze data across multiple groups. Behavioral data was examined using two-way ANOVA with a Bonferroni *post hoc* test, while a t-test was utilized for data between two groups.

## 3 Results

### 3.1 The effect of KF1 on LPS-induced depressive-like behavior in mice

LPS is widely used as a well-established inflammatory factor to induce depressive-like model in mice (Jeon et al., 2017; Sekio and Seki, 2014). Behavioral tests like SPT, OFT, TST, and FST were performed to explore the influence of KF1 on depressive-like behaviors in mice triggered by LPS. As shown in Figure 1, compared with the control group, LPS treatment group decreased the percent of sucrose preference in SPT. However, the treatment with different doses of KF1 (1, 2, 4 mg/kg) and ESC notably reversed the abatement of the percentage of the sucrose (Figure 1B). In the FST and TST tests, the immobility time were increased in LPS-treated mice compared with those in control group, which indicated the depressive and desperate emotion behaviors of mice. By contrast, the treatment with different doses of KF1 (1, 2, 4 mg/kg) and ESC groups remarkably reduced the immobility time in FST and TST tests compared with the LPS treatment group (Figures 1C, D). As expected, the administration of doses of KF1 (1, 2, 4 mg/kg) and ESC also increased the distance traveled in the OFT compared with the LPS treatment (Figure 1E).

### 3.2 KF1 could alleviate the pathological changes of neurons after LPS treatment

**FIGURE 2:**
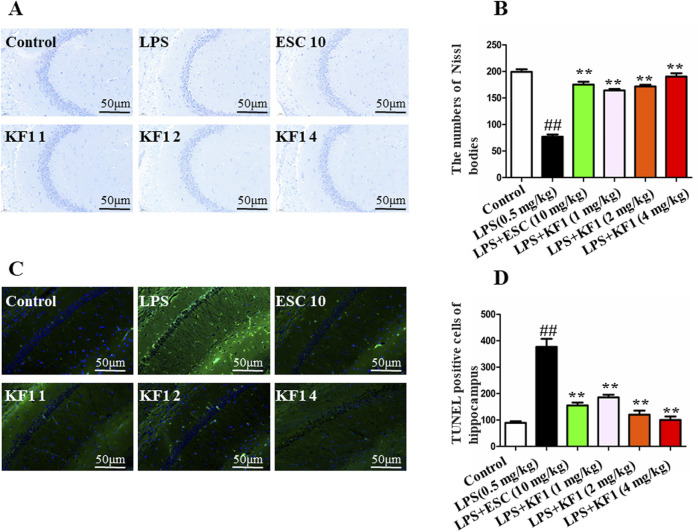
Histopathological changes in the mouse b. **(A)** representative Nissl bodies in the in the hippocampal; **(B)** quantitation of pyramidal cells in the CA1 of hippocampal; **(C)** TUNEL assay for detecting apoptosis in the CA1 of hippocampal; **(D)** Statistical analysis of TUNEL positive cells. The data were expressed as mean ± SEM; ^##^ *p <* 0.01, compared with the control group, **p < 0.01, compare with the LPS group.

The protective effect of KF1 on neuronal morphology in LPS-induced depression-like mice was evaluated using Nissl staining and TUNEL staining. The results of the Nissl staining are shown in Figures 2A, B, control group exhibited normal brain tissue with a round nucleus and a large cell body, no morphological changes were observed. Compared to the control group, the LPS-treated mice displayed marked changes in brain histology, including shrunken cell bodies, nuclear condensation, cell swelling, and interstitial edema. Compared to the LPS group, the treatment with different doses of KF1 (1, 2, 4 mg/kg) and ESC groups mice were remarkably protected, and the number of Nissls body was increased in the hippocampus. This finding suggests that KF1 reduced neuronal damage after different doses of KF1 (1, 2, 4 mg/kg) treatment. TUNEL staining was performed to inspect neuronal apoptosis which green fluorescence indicated apoptotic cells and blue fluorescence marked the nuclei as live cells. As shown in Figures 2C, D, the positive cells had occasional green fluorescence in the control group, whereas the number of apoptosis-positive cells with a high intensity of green fluorescence in the LPS group, indicate that neuronal cells distinct apoptotic features. Additionally, neuronal cells showed a marked decrease in green fluorescence intensity following different doses of KF1 (1, 2, 4 mg/kg) and ESC (10 mg/kg) treatment compared to the LPS group. This suggests that KF1 and ESC therapy provides neuroprotective effects in an LPS-induced depression model.

### 3.3 KF1 reverses the serum and hippocampus cytokine levels

**FIGURE 3:**
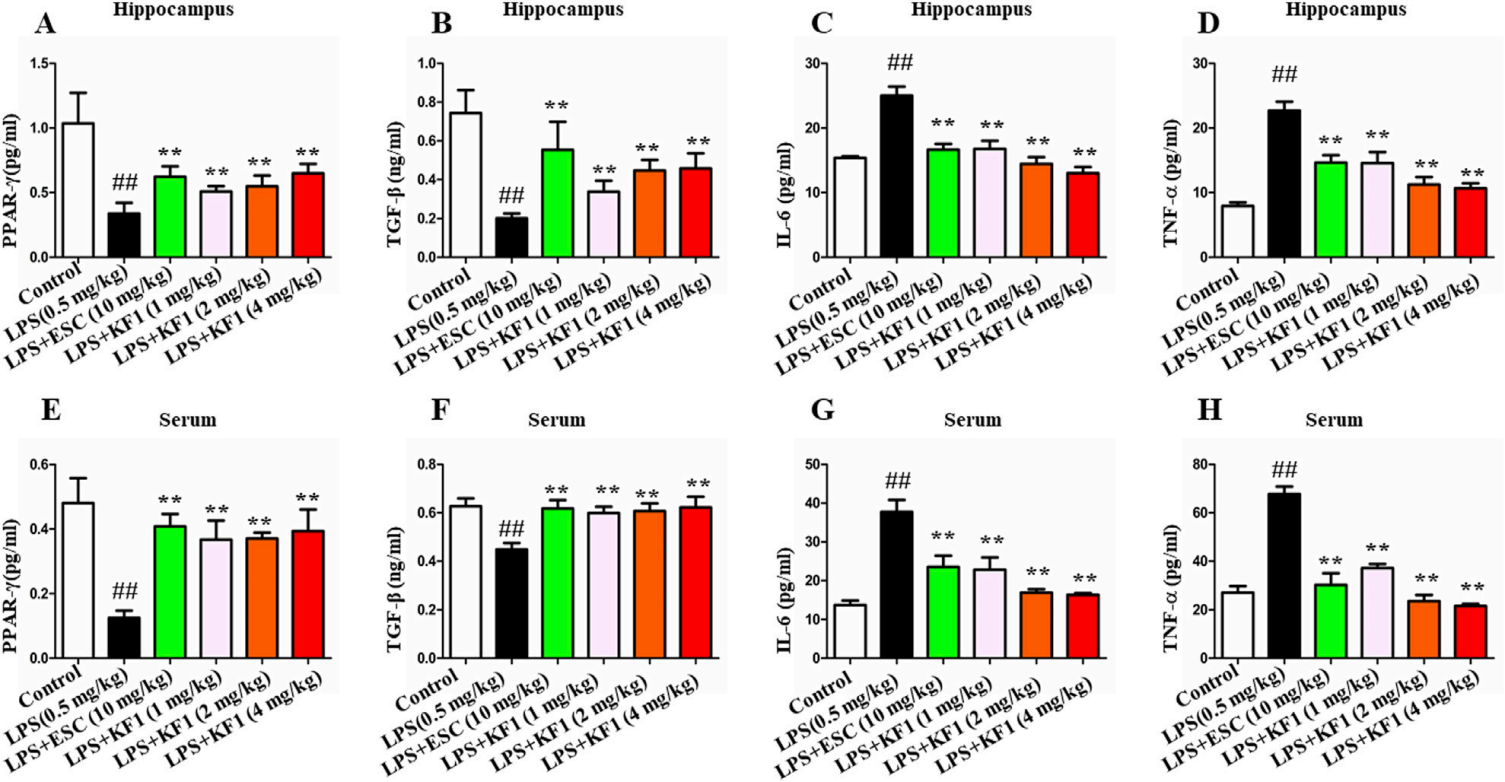
Effects of KF1 on LPS-induced expression of cytokines. **(A–D)** Effects of KF1 on protein levels of PPARγ, TGF-β, IL-6 and TNFα in hippocampus; **(E–H)** Effects of KF1 on protein levels of PPARγ, TGF-β, IL-6 and TNF-α in serum of mice; The data were expressed as mean ± SEM; ^##^ *p <* 0.01, compared with the control group, **p < 0.01, compare with the LPS group.

The cytokines of PPAR-γ,TGF-β, IL-6, and TNFα were tested in the serum and hippocampus of mice using ELISA assay. The findings indicated that LPS notably reduced the protein expressions of PPAR-γ and TGF-β, while elevating TNFα and IL-6 levels in the serum and hippocampus compared to the control group. Of note, different doses of KF1 (1, 2, 4 mg/kg) and ESC (10 mg/kg) treatment markedly increased protein expressions of PPAR-γ and TGF-β, while effectively prevented the LPS induced increase in pro-inflammatory cytokines of TNFα and IL-6 in the serum and hippocampus, when compared to the LPS group (Figure 3).

### 3.4 The effect of KF1 on viability in LPS-induced BV2 cell

**FIGURE 4:**
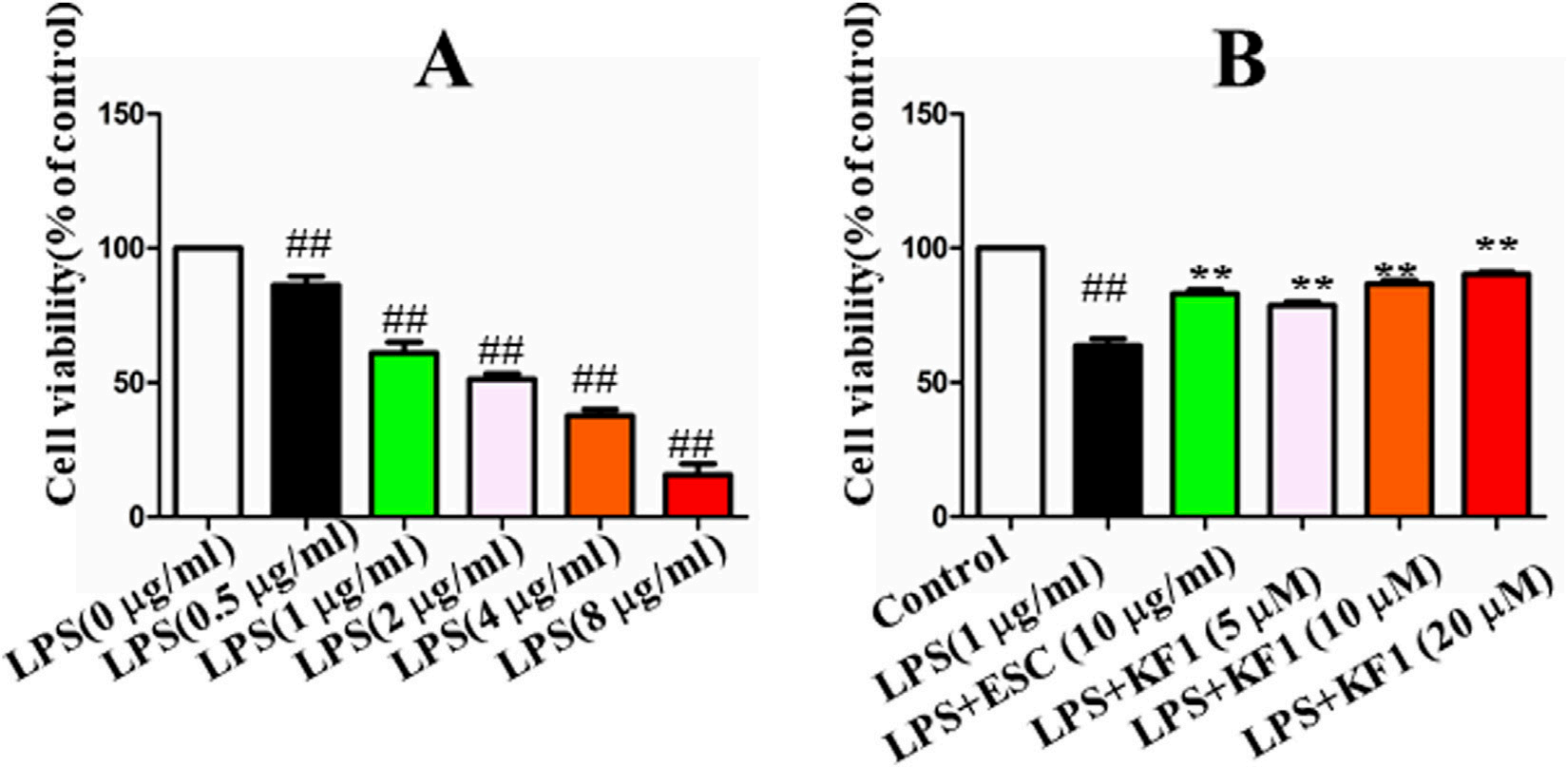
The effect of KF1 on viability in LPS-induced BV2 cell. **(A)** The cell viability of LPS-treated cells after 6 h; **(B)** The protective effect of different concentrations of KF1 were assessed on LPS-induced injury of BV2 cells; The data were expressed as mean ± SEM; ^##^ *p <* 0.01, compared with the control group, **p < 0.01, compare with the LPS group.

To confirm the appropriate concentration of LPS used in BV2 cells, the results showed that LPS 0.5 μg/mL significantly decreased cell survival as shown in Figure 4. After 24 h of LPS treatment, the protective effect of different concentrations of 5 μM, 10 μM, 20 μM KF1 and ESC (10 μg/mL) were assessed on LPS-induced injury of BV2 cells, the results showed that KF1 could dose-dependently increase the LPS-stimulated survival of BV2 cells, indicating the protective effect of KF1 on LPS-stimulated BV2 cells (Figure 4).

### 3.5 KF1 protects LPS -induced BV2 cells apoptosis

**FIGURE 5:**
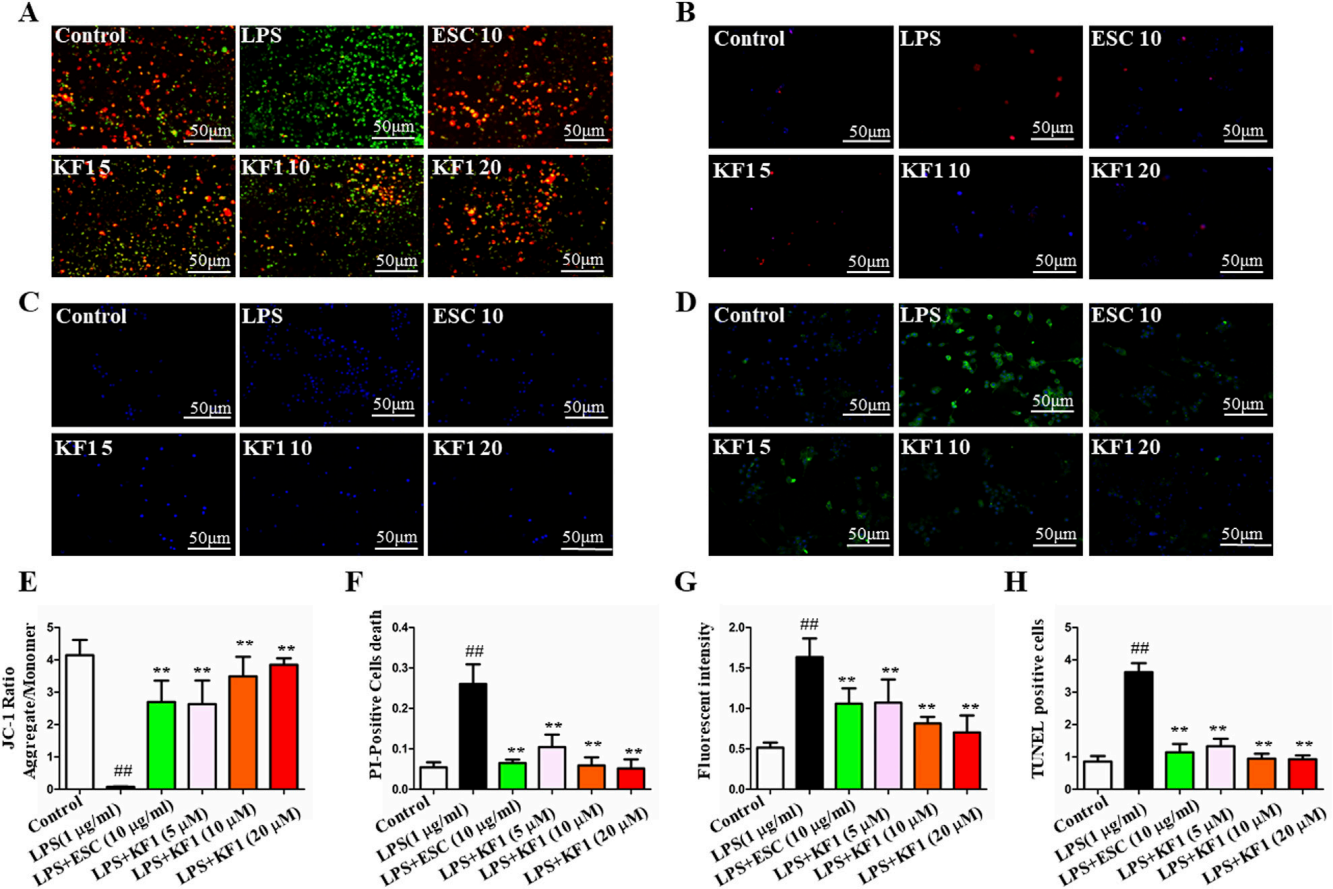
KF1 attenuates LPS-induced mitochondrial injury *in vitro*. **(A)** The fluorescence of neuron cells in each group was observed under a fluorescence microscope after JC-1 staining. **(B)** The apoptosis of neuron cells in each group after PI staining. **(C)** LDH staining. **(D)** TUNEL assay for detecting apoptosis in neurons. **(E)** Statistical analysis comparison of the MMP of neuron cells in different groups. **(F)** Statistical analysis of the PI-positive cells. **(G)** Statistical analysis LDH staining of fluorescent intensity. **(H)** Statistical analysis of TUNEL positive cells. Scale bar, 50 μm. The data were expressed as mean ± SEM; ^##^ *P* < 0.01 compared with the control group, ^**^ *P* < 0.01 compare with the LPS group.

Since apoptosis plays an important role in LPS-induced BV2 cell injury,and mitochondrial dysfunction is one of the key pathological mechanisms of LPS-induced BV2 cell injury, the effect of KF1 on LPS-induced apoptosis in BV2 cells was investigated. The effects of different concentrations of KF1 (5, 10, 20 μM) and ESC (10 μg/mL) on the MMP, PI and LDH were analyzed. The ratio of green fluorescence to red fluorescence represents the degree of mitochondrial depolarization in JC-staining (Liu et al., 2021). Our results showed that the red J-aggregate fluorescence became weaker and the green fluorescence intensity (the ratio of green to red fluorescence) became significantly greater in LPS group. Compared with LPS group, the ratio gradually decreased after treatment with different concentrations of KF1 (5, 10, 20 μM) and ESC (10 μg/mL) in BV2 cells in a dose-dependent manner (Figures 5A, E). Furthermore, PI staining, LDH staining, and TUNEL staining are hallmarks of apoptosis. Our results indicated that the PI staining proportion, LDH levels, and TUNEL positive cells were increased in the LPS group when compared with the control group. However, PI staining proportion (Figures 5B, F), LDH staining fluorescent intensity (Figures 5C, G), and TUNEL positive cells (Figures 5D, H) were significantly decreased in the different concentrations of KF1 (5, 10, 20 μM) and ESC (10 μg/mL) treatment groups compared with in the LPS group, consistent with the aforementioned JC-1 staining result. The above results indicated that apoptosis was significantly inhibited.

### 3.6 KF1 inhibits the effects of LPS on NF-κB/NLRP3 pathway activation *in vivo* and *vitro*

Neuroinflammatory process activated in depression, and LPS may activate the inflammasome via NRLP3 which inflammatory vesicles are critical inflammatory signaling molecular complexes in depression (Li W. et al., 2021; Wang et al., 2018). It is understood that the maturation of IL-1β, which involves the cleavage of Caspase-1, relies on the NLRP3 inflammasome and significantly impacts depression. Consequently, the effects of KF1 on neuroinflammation were studied both *in vivo* and *in vitro*.

**FIGURE 6:**
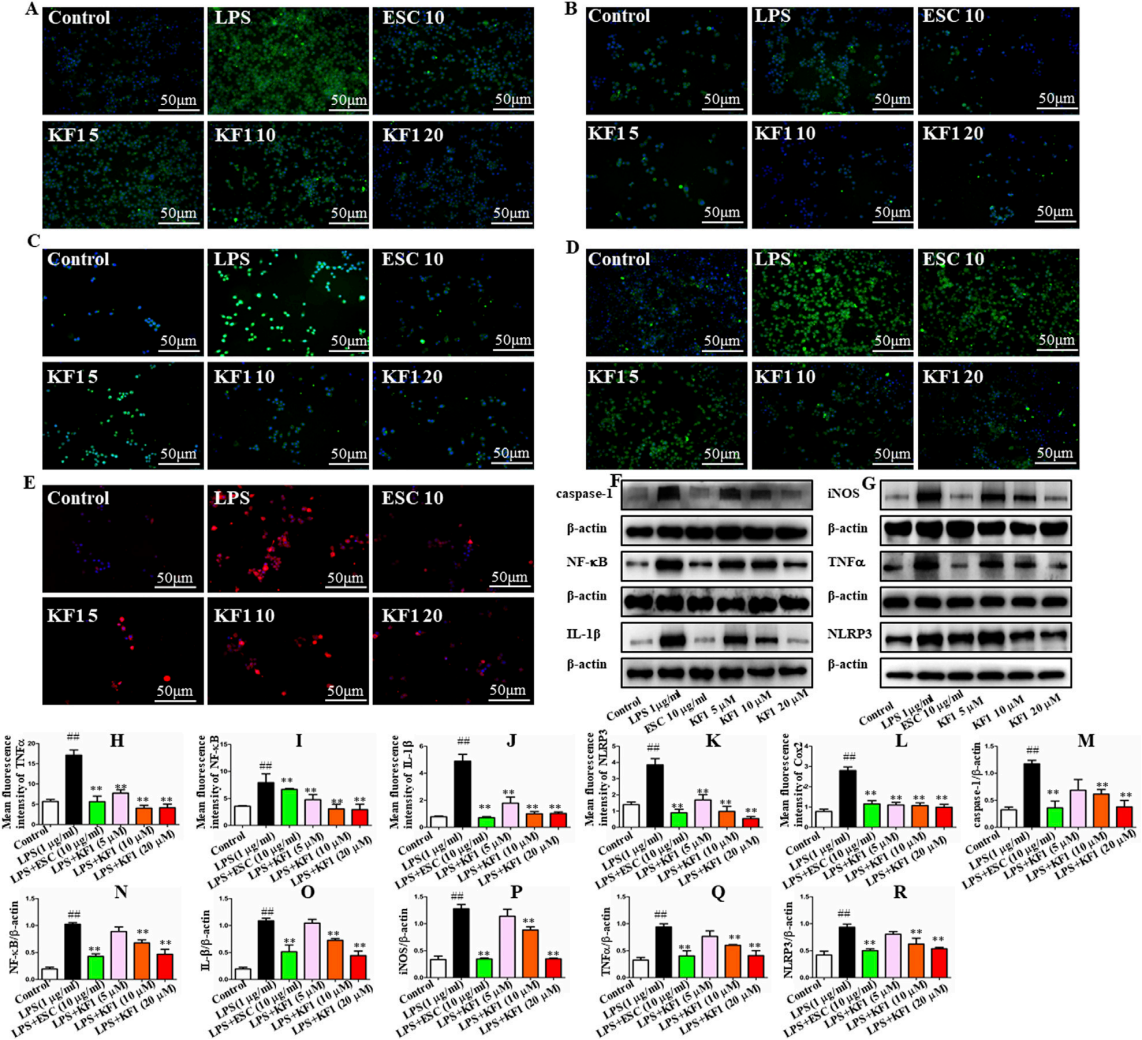
KF1 affects NLRP3/IL-1β pathway activation in BV2 microglia cell lines. **(A–E)** Representative immunofluorescence images of colocalization of Caspase-1, TNFα, NF-κB, IL-1β, and NLRP3 in the BV2 cells. **(F, G)** Representative Western blot analysis of Caspase-1, NF-κB, IL-β, iNOS, TNFα, and NLRP3 protein in the BV2 cell. **(H–L)** The mean fluorescence intensity analysis of TNFα, NF-κB, IL-β, NLRP3, and COX2 by immunofluorescence staining. **(M–R)** Normalized expression of caspase-1, NF-κB, IL-1β, iNOS, TNFα, and NLRP3 protein in the BV2 cells by Western blot analysis. Scale bar, 50 μm. The data were expressed as mean ± SEM; ^##^ *P* < 0.01 compared with the control group, ^**^ *P* < 0.01 compare with the LPS group.

We examined the mean fluorescence intensity and protein expression levels of the downstream NF-κB/NLRP3 inflammasome pathway in BV2 microglial cells by performing immunofluorescence staining and Western blot. As shown in Figure 6, immunofluorescence staining results showed that compared with the control group, the mean fluorescence intensity TNFα, NF-κB, COX2, IL-1β, and NLRP3 in BV2 microglia cells were increased in LPS group. In contrast, different concentrations of KF1 (5, 10, 20 μM) and ESC (10 μg/mL) treatment groups significantly decreased TNFα, NF-κB, COX2, IL-1β, and NLRP3 mean fluorescence intensity compared to the LPS model group. Similar to these results, the proteins expression levels of Caspase-1, NF-κB, IL-1β, iNOS, TNFα, and NLRP3 showed the same trend by Western blot.

**FIGURE 7:**
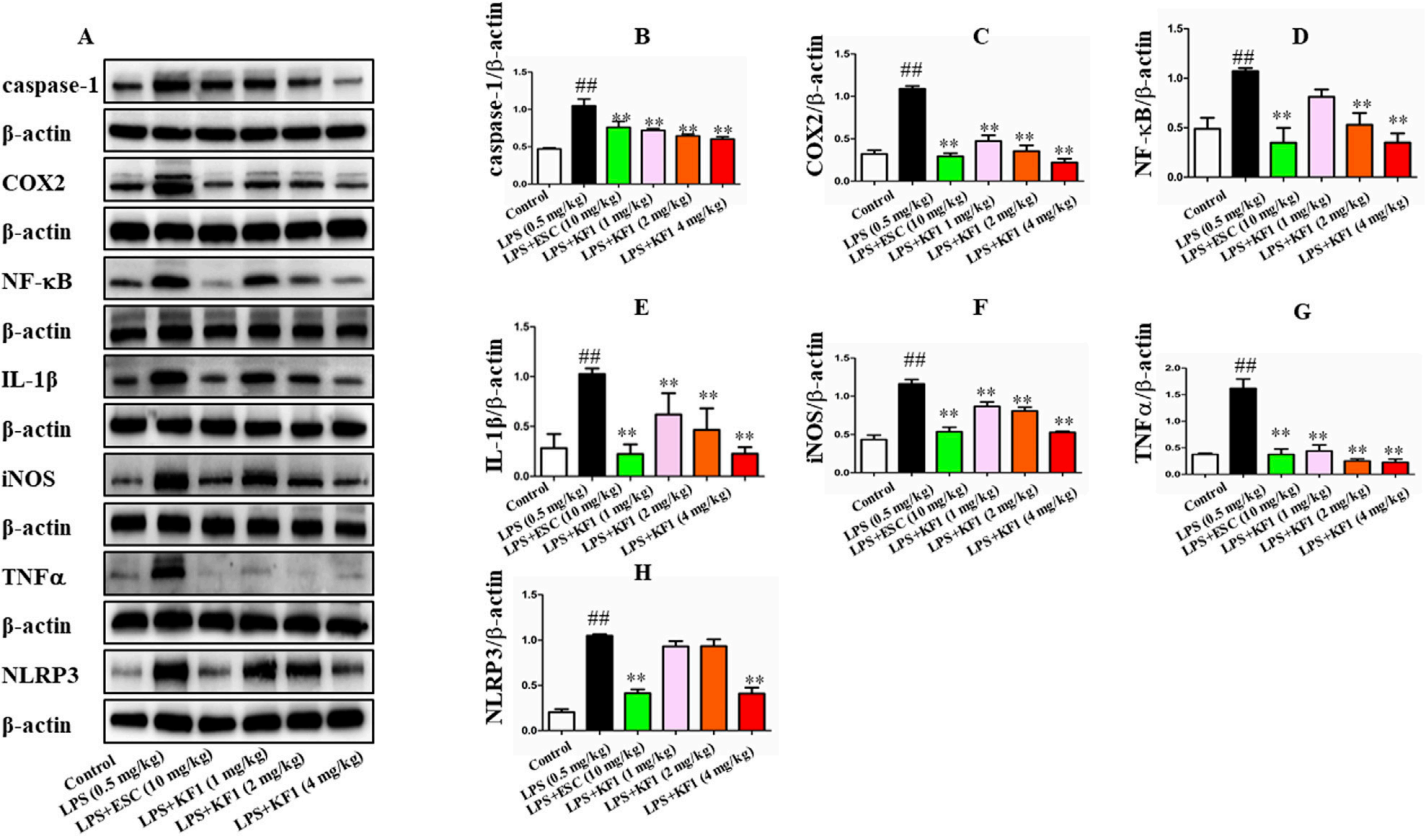
KF1 affects NLRP3/IL-1β pathway activation in LPS-induced mice. **(A)** Representative Western blot analysis of Caspase-1, COX2, NF-κB, IL-1β, iNOS, TNFα, and NLRP3 protein in LPS-induced mice. **(B–H)** The proteins expression of Caspase-1, COX2, NF-κB, IL-1β, iNOS, TNFα, and NLRP3 in LPS-induced mice of hippocampus by Western blot analysis. The data were expressed as mean ± SEM; ^##^ *P* < 0.01 compared with the control group, ^**^ *P* < 0.01 compare with the LPS group.

Next, we proceeded to investigate whether KF1 and ESC inhibits the NF-κB/NLRP3 inflammasome pathway in LPS-induced mice. We observed that LPS induced the protein expression of Caspase-1, COX2, NF-κB, IL-1β, iNOS, TNFα, and NLRP3 were increased in LPS group compared with the control group. However, in comparison with that in LPS group mice, administration of different doses of KF1 (1, 2, 4 mg/kg) and ESC (10 mg/kg) significantly decreased the protein levels of Caspase-1, COX2, NF-κB, IL-1β, iNOS, TNFα, and NLRP3. Taken together, these results indicate that KF1 and ESC antidepressant effects via inhibited the activation of the NLRP3 inflammasome signaling pathways *in vivo* (Figure 7).

### 3.7 KF1 activates PPAR-γ/CX3CR1/Nrf2 expression *in vivo* and *vitro* of LPS-induced model

**FIGURE 8:**
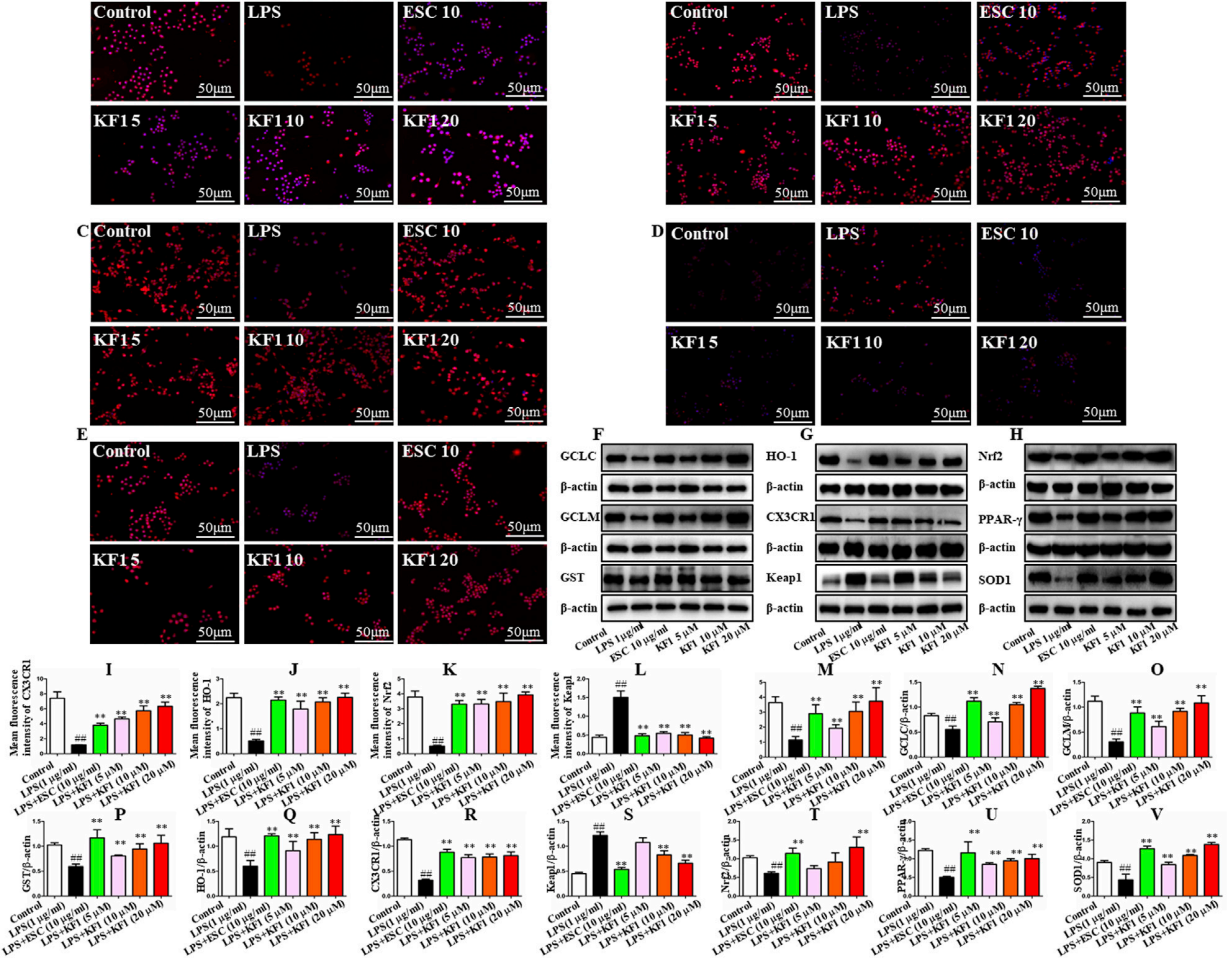
KF1 affects CX3CR1/PPAR-γ/Nrf2 pathway activation in BV2 microglia cell lines. **(A–E)** Representative immunofluorescence images of colocalization of CX3CR1, HO-1, Nrf2, Keap1, and PPARγ in the BV2 cells. **(F–H)** Representative Western blot analysis of GCLC, GCLM, GST, SOD1, HO-1, Nrf2, Keap1, and PPARγ protein in the BV2 cell. **(I–M)** The mean fluorescence intensity analysis of CX3CR1, HO-1, Nrf2, Keap1, and PPARγ by immunofluorescence staining. **(N–V)** Normalized expression of GCLC, GCLM, GST, SOD1, HO-1, Nrf2, Keap1, and PPARγ protein in the BV2 cells by Western blot analysis. Scale bar, 50 μm. The data were expressed as mean ± SEM; ^##^ *P* < 0.01 compared with the control group, ^**^ *P* < 0.01 compare with the LPS group.

PPAR-γ/CX3CR1/Nrf2 is implicated in depression which the key signaling mechanism of oxidative stress (Jiang et al., 2022; Rani and Arya, 2020; Wang B. et al., 2022). We tested the mean fluorescence intensity and protein expression levels of the downstream PPAR-γ/CX3CR1/Nrf2 signaling pathway in BV2 microglial cells by performing immunofluorescence staining and Western blot. As the immunofluorescence staining results described in Figure 8, in comparison to the control group, the mean fluorescence intensity of CX3CR1, HO-1, Nrf2, and PPAR-γ demonstrated significant reduction in LPS group. However, the mean fluorescence intensity of Keap1 was significantly increased in the LPS group compared with the control group. Treatment with different concentrations of KF1 (5, 10, 20 μM) and ESC (10 μg/mL) significantly increased the mean fluorescence intensity of CX3CR1, HO-1, Nrf2, and PPAR-γ compared to the LPS group, nevertheless, the mean fluorescence intensity of Keap1 decreased in treatment with different concentrations of KF1 (5, 10, 20 μM) and ESC (10 μg/mL) groups. Western blot results showed that in the hippocampus of mice, protein levels of GCLC, GCLM, GST, SOD1, HO-1, Nrf2, and PPAR-γ were reduced, while increased protein expression of Keap1 in the LPS group compared to the control group. After treatment with different concentrations of KF1 (5, 10, 20 μM) and ESC (10 μg/mL), Different concentrations of KF1 (5, 10, 20 μM) and ESC (10 μg/mL) significantly enhanced the GCLC, GCLM, GST, SOD1, HO-1, Nrf2, and PPAR-γ protein levels, reduced protein expression of Keap1, compared with the LPS group.

**FIGURE 9:**
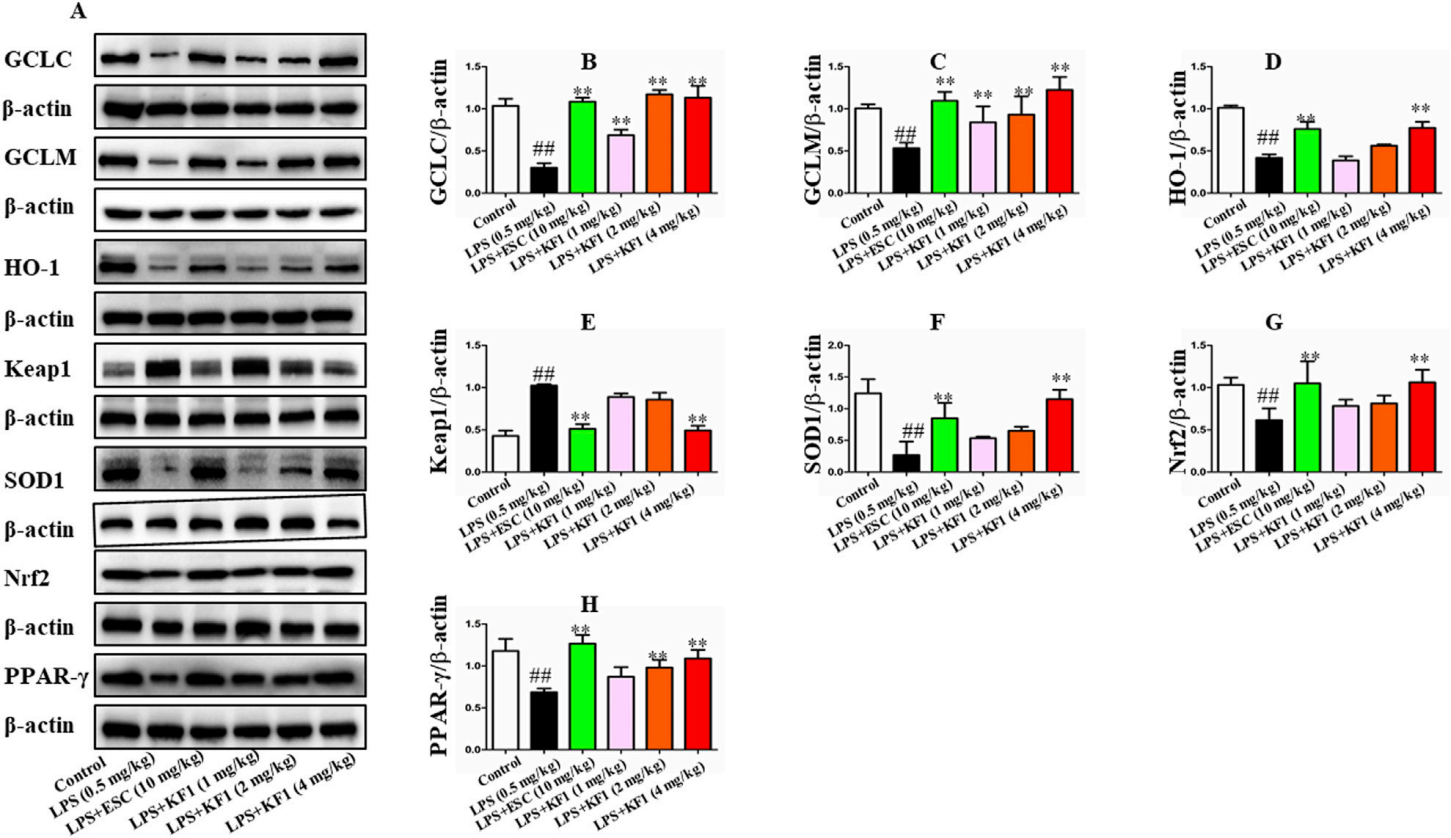
KF1 affects CX3CR1/PPAR-γ/Nrf2 pathway activation in LPS-induced mice. **(A)** Representative Western blot analysis of GCLC, GCLM, SOD1, HO-1, Nrf2, Keap1, and PPARγ protein in LPS-induced mice. **(B–H)** The proteins expression of GCLC, GCLM, SOD1, HO-1, Nrf2, Keap1, and PPARγ in LPS-induced mice of hippocampus by Western blot analysis. The data were expressed as mean ± SEM; ^##^ *P* < 0.01 compared with the control group, ^**^ *P* < 0.01 compare with the LPS group.

In addition, we also examined the protein expressions of GCLC, GCLM, SOD1, HO-1, Nrf2, Keap1, and PPAR-γ in the hippocampus of mice. As shown in Figure 9, the LPS group exhibited a significant decrease in the expressions of GCLC, GCLM, SOD1, HO-1, Nrf2, and PPAR-γ, whereas Keap1 expression was also elevated compared to the control group. In comparison to the LPS group, different doses of KF1 (1, 2, 4 mg/kg) and ESC (10 mg/kg) treatment notably elevated the protein levels of GCLC, GCLM, SOD1, HO-1, Nrf2, and PPAR-γ in the hippocampus of mice, while reducing Keap1 levels.

## 4 Discussion

The present study utilized the LPS-induced depression model in mice and the LPS-stimulated overactivation model in BV2 microglia as research subjects. We found several key observations were made. First, KF1 was able to reverse LPS-induced depressive-like behaviors in mice by SPT, FST, TST, and OFT behavioral experiments, and KF1 restored neurological damage in hippocampal tissues of LPS-induced mice by Nissl staining and TUNEL staining. Second, KF1 attenuates LPS-induced neuroinflammation by inhibiting the release of NLRP3-mediated reduction in pro-inflammatory factor release and containment apoptosis *in vivo* and *in vitro*. Third, KF1 treatment significantly activated the antioxidant signaling pathway triggered by CX3CR1/PPAR-γ/Nrf2 signaling pathway, including altered expression of Nrf2, HO-1, SOD1, PPAR-γ by LPS *in vivo* and *in vitro*.

LPS is a lipophilic molecule that may cross to the brain by blood brain barrier (BBB), and contributes to the production of pro-inflammatory cytokines in the brain and periphery (Kalkman and Feuerbach, 2016). Animal models based on LPS induction is widely used to study for screening antidepressant drug studies (Syed et al., 2018; Yue et al., 2017). The current research assessed the antidepressant effects of KF1 using behavioral evaluations, the most dependable and frequently employed method for screening antidepressant medications, such as SPF, OFT, TST, and FST. Anhedonic-like behavior is a classic symptom of depression, and the SPT is a method to evaluate anhedonic-like behavior in depression animals based on sucrose preference (Shen et al., 2022). The OFT is a classical behavioral experiment used to assess exploratory behavior and behavioral activity in depression animals (Florensa-Zanuy et al., 2021). The TST and FST are mainly used to detect behavioral despair in depressed models (Wang G. et al., 2022). In our study, as expected, mice experienced LPS caused depression-like behaviors in all of these tests, mainly displayed decreased sucrose preference rate in the SPT, reduced total distance moved in the OFT experiment, and increased immobility time in the FST and TST, which were ameliorated by subsequent KF1 treatment, indicating that KF1 was effective in alleviating depression of LPS-induced mice. Furthermore, it has been shown that LPS caused mice displayed heightened depression-like behaviors in the SPT, OFT, FST, and TST tests, which is consistent with our result (Walker et al., 2019; Zhang et al., 2023).

Neuroinflammation plays an important role in CNS disorders, and the association with depression has been widely acknowledged, which is believed to contribute to the initiation, recurrence and worsening of depression (Adzic et al., 2018; Wang et al., 2015). The NLRP3 inflammasome is a multimolecular complex that is thought to be an important molecular platform for the regulation of pro-inflammatory cytokine release. When NLRP3 is activated by stress, it regulates the activation of caspase-1, which in turn promotes the maturation of IL-1β in microglia, and the overproduction of cytokines in microglia contributes to the development of depression (Wang H. et al., 2022). Therefore, when cells are stimulated by LPS a priming step is initiated that activates the NF-κB signaling pathway, leading to activation of inducible NLRP3 (Arioz et al., 2019). The activation of NLRP3 inflammasome promotes the secretion of Caspase-1, which secretes downstream cytokines, NF-κB, TNFα, and other pro-inflammatory cytokines, and then induces apoptosis, which NLRP3 cascade has been implicated CNS disorders such as depression (Beckwith et al., 2020). Our findings indicate that in LPS-induced mice, the levels of TNFα and IL-6 were elevated in serum and hippocampus, and there was an increase in the protein expression of Caspase-1, TNFα, NF-κB, IL-1β, COX2, and NLRP3 in the hippocampus. However, this phenomenon was inhibited by KF1, which is consistent with previously reported findings (Bian et al., 2020). The above results confirmed the inhibitory effect of KF1 on the NF-κB/NLRP3 signaling pathway.

PPAR-γ is a pivotal nuclear receptor, and PPAR-γ agonists may modulate oxidative stress pathways regulating the transcription of various genes involved in cell differentiation, oxidative stress, inflammation, and lipid metabolism (He et al., 2023). PPAR-γ enhances transcription of antiinflammatory and antioxidant genes, and the neuroprotective effects of PPAR-γ activation were investigated in several models of depression (Gold et al., 2013; Shahsavarian et al., 2014). In SHSY5Y cell model, rosiglitazone has a protective effect against mitochondrial dysfunction by increasing the expression of mitochondrial membrane potential, peroxisomal enzymes and SOD and expression, thereby promoting antioxidant defence and inhibiting apoptosis (Jung et al., 2007). CX3CR1 is a signaling pathway that restrains neuroinflammation and modulates the stress response (Picard et al., 2021). Studies have shown that activation of CX3CR1 significantly reduces the release of pro-inflammatory cytokines IL-6, IL-1β, and TNFα, and increases the expression of Nrf2 (Chen et al., 2023). Nrf2 is considered as a regulator of oxidative stress and has emerged as a potential therapeutic target for inflammatory diseases (Robledinos-Antón et al., 2019). Nrf2 translocates into the nucleus and binds to specific DNA sequences, thereby controlling the transcription of downstream molecules. Dysregulation of Nrf2 leads to a decrease in antioxidants and detoxifying enzymes, which has been implicated in the pathogenesis of depression. Once Nrf2 is activated, Keap1 is released from Nrf2 and promotes nuclear translocation of Nrf2, activating the downstream target proteins HO-1, SOD1, GCLC, and GCLM, thus playing a key role in anti-inflammatory and anti-oxidative stress activities of depression. In our study, KF1 treatment significantly restrained the nucleus translocation of Nrf2, prevented the expression of Keap1, enhanced the expression of Nrf2 and its target proteins, including HO-1, SOD1, GCLC, and GCLM *in vivo* and *in vitro,* these results are consistent with previous reports in the literature (Bian et al., 2020; Yang et al., 2023). These findings suggest that KF1 ameliorates LPS-induced depression *in vitro* and *in vivo* mainly through the PPARγ/CX3CR1/Nrf2 pathway.

## 5 Conclusion

To summarise, the present study further supports that LPS-activated inflammasomes accelerate neuroinflammation and lead to depression by triggering PPARγ/CX3CR1/Nrf2 and the NF-κB/NLRP3 signaling pathways. More importantly, the present study discovered that KF1 could reverse the pathological changes in the LPS-induced depression model by activating PPAR-γ/CX3CR1/Nrf2 signaling, and suppressing NF-κB/NLRP3 signaling pathways. These findings provide important theoretical support for KF1 treatment of depression.

## Data Availability

The original data supporting the conclusion of this article will be made available without reservation by the corresponding authors if further enquiry is required.

## Abbreviations

COX2, Cyclooxygenase-2; ESC, Escitalopram; ELISA, Enzyme-linked immunosorbent assay; FST, Forced swimming test; GCLC, Glutamate-cysteine ligase catalytic subunit; GCLM, Glutamate-cysteine ligase modifier subunit; HO-1, Heme oxygenase-1; IL-6, Interleukin-6; IL-1β, Interleukin-1β; iNOS, Inducible nitric oxide synthase; JC-1, Mitochondrial membrane potential dye; KF1, Kaji-ichigoside F1; Keap1, Kelch-like ECH-associated protein 1; LPS, Lipopolysaccharide; LDH, Lactate dehydrogenase; NLRP3, PYD domains-containing protein 3; Nrf2, Nuclear factor erythroid-2-related factor 2; NF-κB, Nuclear factor-κB; OFT, Open field test; PPAR-γ, Peroxisome proliferator-activated receptor γ; PI, Propidium iodide; SOD1, Superoxide dismutase 1; SPT, Sucrose preference test; TST, Tail suspension test; TGF-β, Transforming growth factor-β; TNFα, Tumour necrosis factor-α.
